# Validation of a Dermatology-Focused Multimodal Image-and-Data Assistant in Diagnosis and Management of Common Dermatologic Conditions

**DOI:** 10.3390/medicina62040715

**Published:** 2026-04-09

**Authors:** Joshua Mijares, Emma J. Bisch, Eanna DeGuzman, Kanika Garg, David Pontes, Neil K. Jairath, Vignesh Ramachandran, George Jeha, Andjela Nemcevic, Syril Keena T. Que

**Affiliations:** 1 Department of Dermatology, Indiana University School of Medicine, Indianapolis, IN 46202, USA; 2 Department of Dermatology, Southern Illinois University School of Medicine, Springfield, IL 62702, USA; 3 Department of Dermatology, Philadelphia College of Osteopathic Medicine, Philadelphia, PA 19131, USA; 4 Department of Dermatology, University of Wisconsin, Madison, WI 53706, USA; 5 Skin Institute of New York, New York, NY 10014, USA; 6 Ronald O. Perelman Department of Dermatology, New York University Grossman School of Medicine, New York, NY 10016, USA; 7 Department of Dermatology, Baylor University Medical Center, Dallas, TX 75246, USA; 8 PhDermatology, Tampa, FL 33609, USA

**Keywords:** artificial intelligence, dermatology, multimodal large language model, teledermatology, skin rash, neoplasm, health equity, Fitzpatrick skin tone, digital health, inflammatory skin disease

## Abstract

*Background and Objectives*: Shortages of dermatologists create significant barriers to care, particularly for inflammatory and history-dependent conditions where image-only artificial intelligence (AI) classifiers have limited applicability. Current teledermatology solutions largely focus on single-task, morphology-based neoplasm classifiers, leaving the vast majority of dermatologic presentations underserved. This study evaluated the diagnostic accuracy and management plan quality of Dermflow (Prava Medical, Delaware, USA), a proprietary dermatology-focused Multimodal Image-and-Data Assistant (MIDA) that autonomously gathers dermatology-specific history, integrates data with patient-submitted images, and outputs structured differential diagnoses and management summaries. *Materials and Methods*: Two AI systems, Dermflow and Claude Sonnet 4 (Claude, a leading vision–language model), analyzed 87 clinical images from the Skin Condition Image Network and Diverse Dermatology Images databases, representing 10 inflammatory dermatoses and 9 neoplastic conditions stratified across Fitzpatrick Skin Tone (FST) categories (I–II, III–IV, V–VI). For the diagnostic comparison, Dermflow received images and autonomously gathered clinical history, while Claude received identical images without history. For the management plan comparison, both systems received the correct diagnosis and the clinical histories gathered by Dermflow. The primary outcome was diagnostic accuracy. The secondary outcome was management plan quality, assessed by two blinded dermatologists across eight clinical dimensions using 5-point Likert scales. Chi-square tests compared diagnostic accuracy between models; *t*-tests and ANOVA compared management quality scores. *Results*: Dermflow achieved markedly superior diagnostic accuracy compared to Claude (86.2% vs. 24.1%, *p* < 0.001). Both models maintained consistent diagnostic performance across FST categories without significant within-model differences (Dermflow *p* = 0.924; Claude *p* = 0.828). Management plan quality showed no significant overall differences between models. However, composite management quality scores declined significantly for darker skin tones across both systems: Dermflow scored 4.20 (FST I–II), 3.99 (FST III–IV), and 3.47 (FST V–VI); Claude scored 4.35, 3.97, and 3.44, respectively (*p* < 0.001 for most pairwise FST comparisons within each model). *Conclusions*: Multimodal AI integrating targeted history with image analysis achieves substantially higher diagnostic accuracy than image-only approaches across both inflammatory and neoplastic dermatologic conditions. Autonomous history gathering addresses fundamental limitations of morphology-only classifiers and enables scalable, patient-facing triage across the full spectrum of dermatologic disease. However, both models demonstrated reduced management plan quality for darker skin tones despite receiving the correct diagnosis, suggesting persistent training data limitations that require targeted bias-mitigation strategies beyond domain-specific instruction.

## 1. Introduction

Inflammatory skin diseases represent a substantial global health burden, yet the artificial intelligence (AI) tools developed to support dermatologic care have been disproportionately concentrated on a narrow slice of clinical practice. Systematic reviews consistently demonstrate that dermatology AI literature is dominated by single-task, image-only classifiers for cutaneous neoplasia [1,2,3,4]. This focus leaves the majority of dermatologic presentations underserved: conditions such as eczema, psoriasis, contact dermatitis, and urticaria depend on symptom trajectory, distribution, triggers, and systemic associations that cannot be captured through morphology alone, yet they constitute the bulk of outpatient encounters and teledermatology consultations. Even within their primary domain, image-only classifiers face well-documented limitations, including performance degradation across skin pigmentation and acquisition domains when deployed beyond curated datasets [5,6]. These findings underscore a fundamental vulnerability: pixel-level analysis, however sophisticated, cannot substitute for the clinical reasoning that integrates history, context, and visual findings.

Teledermatology has long been recognized as a critical pathway for expanding access to specialist care, particularly in underserved areas [7,8,9]. However, despite decades of available technology, real-world adoption remains limited, largely restricted to lesion triage rather than the broad spectrum of dermatologic disease [7,10]. The reasons are primarily structural, not technological. Synchronous teledermatology requires real-time clinician availability, patient scheduling, and coordination overhead for encounters that are reimbursed at a fraction of in-person visit rates, making the model economically unsustainable for most practices. Store-and-forward (asynchronous) teledermatology offers a more scalable alternative, but current implementations rely on generic intake forms and patient-submitted images with minimal clinical context [11,12]. These standardized intake tools are not tailored to the presenting condition; for example, a patient with psoriasis receives the same intake as a patient with a suspicious mole, capturing neither the joint symptoms and prior biologic history relevant to the former nor the lesion evolution and family melanoma history relevant to the latter. The result is that the reviewing dermatologist frequently receives an incomplete clinical picture, leading to one of two outcomes: the encounter is escalated to an in-person or synchronous visit (defeating the purpose of asynchronous care), or the clinician initiates iterative back-and-forth messaging to gather missing information (unreimbursed, inefficient, and associated with high patient drop-off) [11,12]. This intake gap is the primary bottleneck limiting the scope and adoption of teledermatology today.

To date, no AI system has addressed this bottleneck by autonomously gathering targeted, condition-adaptive clinical history at the point of patient engagement. Image-only classifiers, by design, cannot collect history. General-purpose multimodal large language models can process both text and images but lack the domain-specific clinical reasoning to know which questions to ask for which presentations [13]. Even recent dermatology-specific foundation models, while demonstrating improved classification performance, remain limited to diagnostic labeling without integrated history collection or management plan generation [14,15]. Medical vision–language models have shown that combining visual and textual inputs can enhance downstream clinical outputs such as report generation and visual question answering [16,17], supporting the premise that integrated signals improve decision quality. Yet none of these systems have been designed as workflow-aware tools that elicit the right clinical information, integrate it with imaging, and produce decision-ready outputs spanning diagnosis and management. This gap persists in part because building such a system requires the intersection of deep dermatologic domain expertise, structured clinical workflow design, and agentic AI architecture, three disciplines that have historically operated in isolation.

These converging challenges, including the narrow scope of existing AI classifiers, the structural workflow limitations of current teledermatology, and the absence of condition-adaptive intake tools, motivate evaluation of systems that move beyond single-task classification toward integrated diagnostic support. Here, we evaluate Dermflow (Prava Medical, Newark, DE, USA), a proprietary Multimodal Image-and-Data Assistant (MIDA) that autonomously gathers targeted, dermatology-specific history, integrates these data with patient-submitted images, and outputs structured differential diagnoses and management summaries for any dermatologic condition. Unlike image-only classifiers or general-purpose language models, Dermflow operates as a complete clinical encounter: it determines which questions are relevant to each patient’s presentation, collects that information through an adaptive conversational interface, and synthesizes history with imaging to produce a structured differential and management plan. To contextualize its performance, we compare Dermflow against Claude Sonnet 4, a state-of-the-art vision–language model representing contemporary image-only medical reasoning capabilities, using diverse clinical images from two validated repositories stratified by Fitzpatrick skin tone to assess both diagnostic accuracy and management plan quality across the spectrum of skin pigmentation. Fairness remains a pressing concern, with documented under-representation of darker skin tones contributing to diagnostic variability across AI systems [6,18], and we specifically evaluate whether multimodal integration mitigates or perpetuates these disparities.

## 2. Materials and Methods

### 2.1. Study Design

This was a retrospective comparative diagnostic accuracy study evaluating the performance of two intake workflows: image submission followed by autonomous history collection (integrated workflow) vs. image submission only (image-only workflow). This study design allowed for assessment of how integration of autonomous history collection in multimodal AI would affect performance. The integrated workflow was completed via a proprietary dermatology MIDA (Prava Medical, Newark, DE, USA), and the image-only workflow was completed via Claude Sonnet 4 (Claude), a leading vision–language model for medical workflows capable of image analysis, in diagnosing common dermatologic conditions across diverse skin tones. The study encompassed both inflammatory dermatoses (rashes) and neoplastic conditions to evaluate multimodal AI performance across the full spectrum of dermatologic presentations.

### 2.2. Image Dataset

Clinical images were obtained from two publicly available, validated repositories with diverse skin tone representation. Sixty images representing 10 inflammatory dermatologic conditions were sourced from the Skin Condition Image Network (SCIN) database [19]: allergic contact dermatitis, eczema, folliculitis, herpes simplex, impetigo, insect bite, irritant contact dermatitis, psoriasis, tinea versicolor, and urticaria. For each rash diagnosis, six images were selected (two per Fitzpatrick Skin Tone [FST] category). Within each FST stratum for rashes, images were further stratified by dermatologist confidence ratings: three images had confidence scores of 1.0 (maximum), while three had scores between 0.55 and 0.67, representing cases with moderate diagnostic uncertainty.

Twenty-seven images representing 9 neoplastic conditions were sourced from the Diverse Dermatology Images (DDI) database [6]: acrochordon, dermatofibroma, Kaposi sarcoma, melanocytic nevus, mycosis fungoides, nevus lipomatosus, seborrheic keratosis, squamous cell carcinoma, and verruca vulgaris. For each neoplasm diagnosis, three images were selected (one per FST category). All 87 images were stratified across three FST categories: FST I–II (lighter skin tones; *n* = 29), FST III–IV (medium skin tones; *n* = 29), and FST V–VI (darker skin tones; *n* = 29).

### 2.3. Clinical Vignettes

Standardized patient histories representative of each diagnosis were developed and presented in a question-and-answer format. These clinical vignettes included chief complaint, lesion duration, evolution over time, attempted treatments, symptoms (including pertinent positives and negatives), alleviating and exacerbating factors, chronic conditions, current medications, allergies, family history, travel history, close contacts, recent exposures, sunscreen use, sun exposure, and psychosocial impact. Identical histories were used across all FST groups for the same diagnosis to ensure consistent clinical context.

### 2.4. AI Model Evaluation

Patient histories were presented to Dermflow in a question-and-answer format simulating an interactive clinical encounter. Dermflow autonomously formulated questions to gather relevant history, and responses were provided only when specifically requested by the system. Dermflow then generated 3 to 5 ranked differential diagnoses for each case.

For the diagnostic accuracy comparison, Claude received only the clinical images without accompanying patient history, demographic information, or clinical context. This intentional image-only approach served as the experimental control, allowing comparison of workflows to assess whether autonomously collected structured history provided diagnostic advantages over image-based evaluation alone. Claude was prompted with the same output instructions coded into Dermflow to ensure a comparable format.

Each model was instructed to output a maximum of 4 differential diagnoses, ranked by likelihood, determined to have greater than 85% likelihood. In addition, each model could provide 1 to 2 additional diagnoses that were potentially life-threatening, highly morbid, rapidly progressive, or involving systemic or other organ involvement (safety diagnoses), even at lower likelihood thresholds.

For the management plan comparison, the correct diagnosis was selected (or provided if absent from the differential), and both models were given the correct diagnosis along with the clinical histories gathered by Dermflow. Both models then generated comprehensive management plans including diagnostic, therapeutic, counseling, and monitoring recommendations. This design placed both systems on equal informational footing for management plan generation, isolating management reasoning from the diagnostic workflow.

### 2.5. Outcome Measures

The primary outcome was diagnostic accuracy, defined as the proportion of cases where the correct diagnosis appeared anywhere within the model’s differential diagnosis list (including both primary likelihood-based diagnoses and safety diagnoses). Accuracy was calculated overall and stratified by FST category.

The secondary outcome was management plan quality, assessed by two independent, board-certified dermatologists who were blinded to the AI model source. Reviewers evaluated each management plan using eight 5-point Likert scale items assessing clinical appropriateness, safety, clinical utility, screening appropriateness, diagnostic workup quality, treatment plan quality, counseling adequacy, and monitoring plan appropriateness (Table 1). More details regarding scoring criteria can be found in Table S1. Scores of 1 indicated poor or inappropriate quality, and scores of 5 indicated excellent quality. Composite scores were calculated as the mean across all eight domains.

### 2.6. Statistical Analysis

Diagnostic accuracy was compared between models using McNemar’s exact test for paired proportions, as each image was evaluated by both systems. Accuracy rates are reported as percentages with 95% confidence intervals calculated using the normal approximation method. Management plan quality scores were compared using independent samples *t*-tests for overall comparisons and analysis of variance (ANOVA) with Tukey post hoc testing for FST-stratified analyses. Two-sided *p*-values less than 0.05 were considered statistically significant. To control for multiple comparisons across 59 hypothesis tests, the Benjamini–Hochberg procedure was applied with a false discovery rate of 5%. Post hoc power analysis using an exact McNemar framework confirmed adequate statistical power for the primary outcome (see Supplementary Protocol for complete power analyses). All analyses were performed using R version 4.5.1 [20], and figures were generated with the ggplot2 package version 4.0.0 [21]. Evaluations using Claude were completed in July 2025, using the most current Claude Sonnet 4 model available at that time.

## 3. Results

### 3.1. Diagnostic Performance

Dermflow achieved markedly superior diagnostic accuracy compared to Claude across all diagnoses. Overall, Dermflow correctly identified the diagnosis in 86.2% of cases (95% CI: 77.1–92.7%) versus 24.1% for Claude (95% CI: 15.6–34.5%), representing an absolute difference of 62.1 percentage points (*p* < 0.001, McNemar’s exact test; Table 2). Among the 87 paired image evaluations, Dermflow correctly diagnosed 55 cases that Claude missed, while Claude correctly diagnosed only 1 case that Dermflow missed (discordant pair ratio 55:1).

Both models maintained consistent diagnostic performance across FST categories without statistically significant within-model differences. Dermflow achieved accuracy rates of 86.2% (FST I–II), 89.7% (FST III–IV), and 82.8% (FST V–VI; *p* = 0.924). Claude achieved 24.1% (FST I–II), 20.7% (FST III–IV), and 27.6% (FST V–VI; *p* = 0.828; Table 2).

When examined by condition category, the pattern was consistent. For inflammatory dermatoses, Dermflow achieved 88.3% accuracy (95% CI: 80.2–96.5%) versus Claude’s 21.7% (95% CI: 11.2–32.1%; *p* < 0.001). For neoplastic conditions, Dermflow achieved 81.5% accuracy (95% CI: 65.8–97.1%) versus Claude’s 29.6% (95% CI: 11.2–48.0%; *p* < 0.001). Neither condition category showed significant within-model FST differences (all *p* > 0.05).

### 3.2. Management Plan Quality

**Figure 1:**
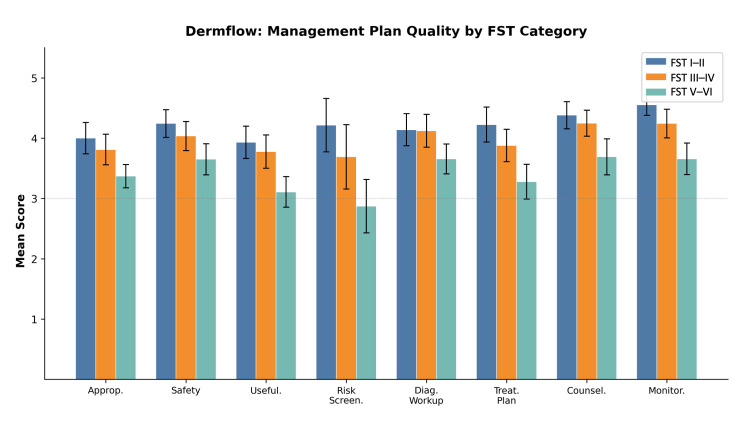
Management plan quality scores of Dermflow, stratified by Fitzpatrick Skin Tone category. Error bars represent 95% confidence intervals.

**Figure 2:**
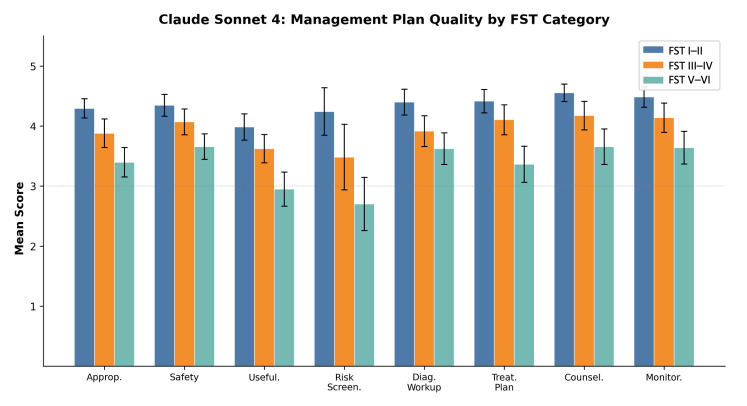
Management plan quality scores of Claude Sonnet 4, stratified by Fitzpatrick Skin Tone category. Error bars represent 95% confidence intervals.

Overall management plan quality scores showed no significant differences between Dermflow and Claude across all eight clinical dimensions (Table 3). However, both models demonstrated significant degradation in management quality for darker skin tones across all clinical dimensions (Table 4; Figure 1 and Figure 2).

Composite management quality scores declined progressively with darker skin: Dermflow scored 4.20 (95% CI: 4.01–4.39) for FST I–II, 3.99 (95% CI: 3.79–4.19) for FST III–IV, and 3.47 (95% CI: 3.28–3.65) for FST V–VI. Claude scored 4.35 (95% CI: 4.22–4.48), 3.97 (95% CI: 3.77–4.16), and 3.44 (95% CI: 3.22–3.66) for FST I–II, III–IV, and V–VI, respectively. Pairwise FST comparisons within each model were significant for FST III–IV vs. V–VI and FST I–II vs. V–VI (all *p* < 0.001); the FST I–II vs. III–IV comparison reached significance for Claude (*p* = 0.002) but not for Dermflow (*p* = 0.143). No significant differences were observed between models at any FST level (all *p* > 0.05). Both reviewers demonstrated the same directional pattern of declining scores with darker FST categories, supporting the reliability of the observed FST-related trends despite moderate absolute agreement.

The FST-related management quality degradation was driven primarily by the inflammatory dermatosis cases. For neoplastic conditions analyzed separately, no significant degradation in management quality was observed across FST categories for either model (see Supplementary Figures S1–S4).

## 4. Discussion

### 4.1. Diagnostic Accuracy

Dermflow achieved substantially superior diagnostic accuracy compared to Claude (86.2% vs. 24.1%, *p* < 0.001), demonstrating the value of integrating targeted history collection with image analysis. This 62-percentage-point difference is clinically significant. The paired analysis is particularly informative: of 87 images evaluated by both systems, Dermflow correctly diagnosed 55 cases that Claude missed, while Claude outperformed Dermflow on only a single case. This 55:1 discordant pair ratio indicates that the accuracy gap is not driven by a small subset of diagnoses or chance variation; instead, it reflects a consistent, systematic advantage across the range of clinical presentations evaluated. The finding aligns with established principles in medical AI, where domain-specific systems consistently outperform general-purpose architectures [1,14,15]. The diagnostic gold standard in this study was the human-assigned diagnosis from each image database, ensuring that accuracy reflects agreement with expert clinical assessment rather than an algorithmic reference.

An anticipated critique of this comparison is that it demonstrates only that clinical history improves diagnosis, a finding that would surprise no clinician. This observation, while technically correct, misses the more consequential point. The question was never whether history matters. The question was whether an AI system could autonomously gather the right history for each clinical scenario and integrate it with imaging to produce actionable outputs. Current teledermatology platforms use generic, static intake forms that capture the same information regardless of the presenting condition. A dermatologist evaluating psoriasis needs joint symptoms, prior biologic use, and body surface area estimates. A dermatologist evaluating a changing mole needs lesion evolution timeline, sun exposure history, and family melanoma history. No existing intake tool adapts to the clinical presentation, and no prior AI system has been designed to fill this role. The diagnostic accuracy gap observed here reflects not just the value of history, but the value of condition-specific history, gathered systematically and integrated with imaging in a structured workflow.

The clinical significance of specific history elements varied by diagnosis. For conditions such as Kaposi sarcoma, information about HIV status or immunosuppressive therapy is diagnostically decisive, as morphologically similar lesions in an immunocompetent patient, such as bacillary angiomatosis, carry different prior probabilities than the same lesions in a patient with advanced HIV. This case-level dependence on targeted history emphasizes why condition-adaptive intake, which asks about HIV status for suspected vascular lesions but not for eczema, is fundamentally different from generic intake forms that apply the same questions to every patient.

The study design provides internal evidence that the diagnostic accuracy gap is attributable to the history-gathering workflow rather than to differences in underlying model quality. For the management plan comparison, Claude received both the correct diagnosis and the clinical histories that Dermflow had gathered, placing both systems on equal informational footing. Under these conditions, Claude performed equivalently to Dermflow across all eight management quality dimensions (Table 3). This result confirms that the underlying model architecture is comparably capable when given the same inputs. The 62-percentage-point diagnostic accuracy gap therefore reflects the specific contribution of Dermflow’s autonomous, condition-adaptive history-gathering workflow, not a generalized model superiority.

The diagnostic performance gap was nearly as large for neoplastic conditions (81.5% vs. 29.6%) as for inflammatory dermatoses (88.3% vs. 21.7%). For neoplastic conditions, where visual morphology is traditionally considered paramount, one might expect image-only analysis to perform more competitively. That it did not suggests that clinical context (lesion evolution, sun exposure, immunosuppression status, personal and family cancer history) enhances diagnostic reasoning even for morphology-prominent presentations, reinforcing the rationale for multimodal approaches across all of dermatology.

Both models maintained equitable diagnostic performance across FST categories. Dermflow achieved consistent accuracy regardless of skin pigmentation (86.2%, 89.7%, and 82.8% for FST I–II, III–IV, and V–VI; *p* = 0.924), as did Claude within its lower performance range. This finding is particularly relevant in the context of prior literature documenting significant performance degradation for darker skin tones in image-only classifiers [5,6]. That degradation is an inherent vulnerability of morphology-only pipelines, which depend entirely on visual features that vary with skin pigmentation. The multimodal approach evaluated here reduces this structural dependence by grounding diagnostic reasoning in clinical history alongside imaging. When a system knows that a patient has a family history of melanoma, rapid lesion growth, and immunosuppression, the diagnostic weight shifts from pixel-level pattern matching toward clinical probability, which does not vary with skin tone. This mechanism may explain why Dermflow achieved consistent accuracy across all FST categories and suggests that autonomous history gathering is not only a workflow improvement but also an equity intervention.

### 4.2. Management Plan Quality and the Equity Gap

As noted above, the absence of significant overall differences in management quality between Dermflow and Claude is consistent with the study design as both systems received the correct diagnosis and the same clinical history before generating management plans, isolating management reasoning from the diagnostic workflow. This equivalence serves as an important internal control, confirming that management quality differences are not driven by the system itself but by other factors.

The significant and consistent decline in management quality for darker skin tones across both systems is the more consequential finding. Composite scores declined from 4.20 to 3.47 (Dermflow) and 4.35 to 3.44 (Claude) between FST I–II and FST V–VI, with most pairwise FST comparisons reaching statistical significance. This pattern persisted across all eight clinical dimensions, suggesting a systematic rather than domain-specific limitation. Critically, this degradation occurred despite both models receiving the correct diagnosis prior to management plan generation, indicating that the disparity operates at the level of therapeutic reasoning rather than diagnostic performance. One nuance warrants attention: for Dermflow, the decline in composite management quality between FST I–II and FST III–IV did not reach statistical significance (*p* = 0.143), while the analogous comparison for Claude was significant (*p* = 0.002). This suggests that Dermflow may exhibit a somewhat more gradual onset of FST-related management degradation compared to Claude, though both systems showed a significant decline between FST III–IV and FST V–VI. The observation that neoplastic conditions showed no significant FST-related management degradation, while inflammatory conditions drove the observed disparity, suggests that the underlying training data may be differentially limited in representing inflammatory disease management across diverse skin tones.

Several potential explanations deserve consideration. First, neoplastic management guidelines are relatively standardized across populations, including excision margins, surveillance intervals, and referral thresholds. On the other hand, inflammatory disease management is more contextually variable and population-specific. Treatment selection for psoriasis, atopic dermatitis, and other chronic inflammatory conditions may appropriately differ across skin tones: phototherapy response varies with baseline pigmentation and carries differential hyperpigmentation risk. Training data for large language models may underrepresent clinical guidelines, case reports, and therapeutic literature addressing inflammatory skin disease management in patients with darker skin, meaning that both models may have encountered fewer high-quality examples of condition-specific inflammatory management tailored to patients with FST V–VI. Second, the observed pattern could partially reflect reviewer-side effects as dermatologists may apply heightened scrutiny to management recommendations for underrepresented populations, reflecting awareness of historical care inequities. Disentangling these contributions will require prospective studies with objective outcome measures rather than subjective quality ratings.

### 4.3. Implications for Clinical Practice and Teledermatology

These findings speak directly to the structural barriers limiting teledermatology adoption. The economic reality of synchronous teledermatology (real-time clinician availability, scheduling coordination, and reimbursement rates that are often a fraction of in-person visit rates) has rendered that model unsustainable for most dermatology practices. Asynchronous store-and-forward teledermatology is the more scalable pathway, but its adoption has been constrained not by the absence of imaging AI, but by the absence of adequate clinical intake. When a dermatologist receives an asynchronous packet containing photos and a generic intake form, the information is rarely sufficient for a confident diagnostic and management decision. The encounter is then either escalated to an in-person visit, defeating the purpose of asynchronous care, or the clinician initiates unreimbursed back-and-forth messaging that is inefficient and from which patients frequently disengage.

The multimodal approach evaluated here addresses this bottleneck directly. By autonomously gathering condition-adaptive history at the point of patient engagement, the system produces a decision-ready clinical encounter rather than a partial intake requiring clinician intervention. The 86.2% diagnostic accuracy achieved with this workflow suggests that many cases could be resolved asynchronously without the scheduling overhead, coordination burden, and reimbursement penalties of synchronous encounters. For inflammatory conditions, which constitute most outpatient dermatologic presentations but have been almost entirely excluded from the scope of existing AI tools and conventional teledermatology platforms, this represents a meaningful expansion of what asynchronous dermatologic care can achieve.

However, the equity gap in management quality underscores that diagnostic accuracy alone is not sufficient. A system that correctly identifies a condition but generates lower-quality management plans for certain patient populations does not deliver equitable care. This finding highlights the need for targeted bias-mitigation strategies, including curated training data representing diverse populations, equity-aware instruction sets, and systematic bias auditing, that go beyond general domain expertise. The magnitude of improvement achieved through domain-specific instruction for diagnostic accuracy (over 60 percentage points) suggests that similarly targeted interventions for management reasoning could address the observed disparities. More broadly, the integration of Internet of Things (IoT) devices, including wearable sensors, connected cameras, and environmental monitors, into teledermatology could further enrich the multimodal inputs that systems like Dermflow are designed to integrate to provide more patient-specific outputs.

### 4.4. Limitations

Several limitations warrant discussion. First, while Dermflow autonomously gathered clinical history, the histories themselves were constructed from standardized patient vignettes rather than real patient interactions. This represents an idealized scenario in which the patient provides complete, consistent, and accurate responses. Additionally, the standardized vignettes used in this study were composed by clinicians using precise medical terminology; real patients presenting through a teledermatology interface would use lay language that may be less systematically organized. Prior work has demonstrated that the phrasing and medical precision of patient-provided history substantively influence model outputs in clinical AI applications [22]. In real-world deployment, patients may provide incomplete or tangential information, and the system’s ability to elicit usable history from diverse patient populations with varying health literacy remains untested. Dermflow’s adaptive history-gathering workflow mitigates this limitation partially by asking targeted questions rather than relying on patient-generated free text, but prospective studies with real patient-system encounters are essential to validate this component of the workflow.

Second, a post hoc technical review identified that Dermflow processed a truncated subset of the clinical history it gathered (approximately 500 characters) due to a software limitation during the evaluation period. The reported diagnostic accuracy therefore reflects performance under suboptimal input conditions; full utilization of the gathered clinical history would be expected to further improve diagnostic performance, suggesting that the 86.2% accuracy reported here represents a performance floor rather than the system’s full capability.

Third, the diagnostic gold standard was the expert-assigned diagnosis from each database rather than histopathologic confirmation for all cases. While the SCIN and DDI databases use expert dermatologist annotation, some degree of diagnostic uncertainty is inherent in any image-based reference standard.

Fourth, management plan quality was assessed by two blinded dermatologists using Likert scales, and inter-rater reliability metrics (such as Cohen’s kappa or intraclass correlation coefficients) should be computed from the raw scoring data and reported to strengthen interpretability. Future studies should include formal reliability analyses and consider larger reviewer panels.

Fifth, inflammatory and neoplastic images were drawn from two different databases (SCIN and DDI, respectively), which introduces potential variation in image quality, annotation methodology, and metadata standards. This was a necessary design choice as no single publicly available database contains adequately diverse representation of both inflammatory and neoplastic conditions across the full FST spectrum. The use of two complementary, validated repositories was the best available approach to achieving the clinical and demographic breadth required for this evaluation, but cross-category comparisons should be interpreted with this caveat. Additionally, the 19 diagnoses included span a wide range of intrinsic diagnostic difficulty. This heterogeneity was inherent to the study design, which sought to evaluate performance across the clinical spectrum rather than on a uniformly challenging or uniformly straightforward case set.

Sixth, the sample size of 87 images across 19 diagnoses, while adequate for the primary outcome based on post hoc power analysis (see Supplementary Protocol), limits statistical power for per-diagnosis subgroup analyses. Seventh, Dermflow is a proprietary system; therefore, while its clinical workflow and evaluation methodology are fully described, the internal prompt architecture and instruction sets are not publicly available, consistent with standard practice for commercial AI systems in validation studies.

Eighth, the retrospective design with curated research images may not reflect the variability of patient-submitted photographs in real-world teledermatology, where image quality, lighting, and framing are uncontrolled. Taken together, the use of standardized vignettes, curated research images, and controlled input conditions means that reported accuracy and management quality figures should be interpreted as estimates of best-case performance under idealized conditions, rather than predictions of real-world deployment performance. Prospective studies with live patient interactions and uncontrolled image conditions are required before clinical implementation.

Finally, the model was not instructed to evaluate validated and objective tools to assess severity and staging assessment, such as the Eczema Area and Severity Index (EASI) and the Investigator’s Global Assessment (IGA), which are often employed in inflammatory dermatoses to guide treatment and biologic eligibility.

### 4.5. Future Directions

Technical optimization of Dermflow’s history integration pipeline, including resolution of the truncation limitation identified in this study, is expected to yield further accuracy improvements and represents an immediate development priority. Concurrently, the most important research priority is prospective validation of Dermflow’s autonomous history-gathering workflow with real patients in clinical teledermatology settings, in which Dermflow replaces or supplements the intake process for new dermatology referrals. Key implementation metrics would include the proportion of encounters resolved without escalation, time from patient submission to clinical decision, patient engagement rates with the adaptive history interface (particularly across age, health literacy, and language groups), completeness of patient-provided responses, and dermatologist-rated confidence in the Dermflow-generated encounter packet compared to standard intake. These workflow efficiency metrics are ultimately more relevant to real-world adoption than diagnostic accuracy in isolation.

Second, future studies should include a clinician comparator arm. The 86.2% accuracy observed here is encouraging, but it requires benchmarking against dermatologist performance on the same cases with the same information to determine whether this level of accuracy is sufficient for safe clinical deployment as a triage or decision-support tool.

Third, addressing the identified management quality disparities across skin tones is critical. Targeted instruction-based interventions, including explicit equity review protocols, curated reference datasets representing inflammatory disease management across diverse populations, and systematic bias auditing, should be developed and evaluated. The magnitude of improvement achieved through domain-specific instruction for diagnostic accuracy provides a strong rationale for similar approaches to management reasoning.

Fourth, integration of structured severity scoring, such as the EASI and IGA, into multimodal AI workflows is a meaningful next step, as these tools depend on both morphologic assessment and clinical history. Finally, expanding evaluation to include additional AI systems, larger and more diverse datasets, formal inter-rater reliability reporting, and direct cost-effectiveness comparisons with existing teledermatology models will be essential to translating these findings into clinical practice.

## 5. Conclusions

Multimodal AI that autonomously gathers targeted, condition-adaptive clinical history and integrates it with image analysis achieves substantially higher diagnostic accuracy than image-only approaches for both inflammatory and neoplastic dermatologic conditions, with equitable diagnostic performance across skin tones. These findings suggest that the primary barrier to scalable asynchronous teledermatology is not the absence of better imaging classifiers, but the absence of intelligent clinical intake that produces decision-ready encounters. By addressing this intake gap, multimodal systems have the potential to expand the scope of AI-augmented teledermatology beyond lesion triage to the full spectrum of dermatologic disease. However, both systems demonstrated reduced management quality for patients with darker skin tones, highlighting persistent equity challenges in therapeutic reasoning that must be addressed through targeted bias-mitigation strategies and investment in diverse training data.

## Figures and Tables

**Table 1:** Questions and examples considered by dermatologist reviewers when rating management plans.

Rating Category	Question
Appropriateness	How clinically appropriate is this management plan?
Safety	How safe is this management plan for the patient?
Usefulness	How useful would this management plan be for a dermatologist in clinical practice?
Risk Screening	How well does the management plan account for appropriate screening of associated conditions (if applicable)?
Diagnostic Workup	Overall rating of AI diagnostic workup plan.
Treatment Plan	Overall rating of AI treatment plan.
Counseling	Overall rating of AI counseling.
Monitoring	Overall rating of AI monitoring plan.

**Table 2:** Diagnostic accuracy by model and Fitzpatrick Skin Tone (FST) category.

	Dermflow, % (95% CI)	Claude, % (95% CI)	*p*-Value *
Overall	86.2 (77.1–92.7)	24.1 (15.6–34.5)	<0.001
FST I–II	86.2 (68.3–96.1)	24.1 (10.3–43.5)	<0.001
FST III–IV	89.7 (72.6–97.8)	20.7 (8.0–39.7)	<0.001
FST V–VI	82.8 (64.2–94.2)	27.6 (12.7–47.2)	<0.001
*p*-Value †	0.924	0.828	—

* Between-model comparison. † Within-model comparison across FST categories.

**Table 3:** Overall management plan quality scores by model (mean ± SEM).

Rating Dimension	Dermflow	Claude	*p*-Value
Appropriateness	3.73 ± 0.07	3.86 ± 0.07	0.203
Safety	3.98 ± 0.07	4.02 ± 0.06	0.635
Usefulness	3.61 ± 0.08	3.52 ± 0.08	0.416
Risk Screening	3.57 ± 0.15	3.43 ± 0.15	0.512
Diagnostic Workup	3.97 ± 0.08	3.98 ± 0.08	0.958
Treatment Plan	3.79 ± 0.09	3.96 ± 0.08	0.161
Counseling	4.10 ± 0.08	4.13 ± 0.07	0.834
Monitoring	4.15 ± 0.07	4.09 ± 0.07	0.539

**Table 4:** Composite mean management plan scores by model and FST category (mean, 95% CI).

	Dermflow	Claude	*p*-Value *
Overall	3.89 (3.77–4.00)	3.92 (3.80–4.04)	0.770
FST I–II	4.20 (4.01–4.39)	4.35 (4.22–4.48)	0.205
FST III–IV	3.99 (3.79–4.19)	3.97 (3.77–4.16)	0.868
FST V–VI	3.47 (3.28–3.65)	3.44 (3.22–3.66)	0.864
*p* (I–II vs. III–IV)	0.143	0.002	—
*p* (III–IV vs. V–VI)	<0.001	<0.001	—
*p* (I–II vs. V–VI)	<0.001	<0.001	—

* Between-model comparison at each FST level.

## Data Availability

The datasets generated and analyzed during the current study are available from the corresponding author on reasonable request, subject to confidentiality restrictions protecting proprietary system elements during technology transfer and commercialization processes.

## Supplementary Materials

The following supporting information can be downloaded at: https://www.mdpi.com/article/10.3390/medicina62040715/s1, Supplementary Protocol (complete study protocol including power analyses); Figure S1: Management plan quality scores of Dermflow for inflammatory dermatoses, stratified by Fitzpatrick Skin Tone category. Error bars represent 95% confidence intervals; Figure S2: Management plan quality scores of Claude Sonnet 4 for inflammatory dermatoses, stratified by Fitzpatrick Skin Tone category. Error bars represent 95% confidence intervals; Figure S3: Management plan quality scores of Dermflow for neoplastic conditions, stratified by Fitzpatrick Skin Tone category. Error bars represent 95% confidence intervals; Figure S4: Management plan quality scores of Claude Sonnet 4 for neoplastic conditions, stratified by Fitzpatrick Skin Tone category. Error bars represent 95% confidence intervals; Table S1: Questions and examples considered by dermatologist reviewers when rating management plans.

## References

[1] Jairath N., Pahalyants V., Shah R., Weed J., Carucci J.A., Criscito M.C. (2024). Artificial Intelligence in Dermatology: A Systematic Review of Its Applications in Melanoma and Keratinocyte Carcinoma Diagnosis. Dermatol. Surg..

[2] Salinas M.P., Sepúlveda J., Hidalgo L., Peirano D., Morel M., Uribe P., Rotemberg V., Briones J., Mery D., Navarrete-Dechent C. (2024). A Systematic Review and Meta-Analysis of Artificial Intelligence versus Clinicians for Skin Cancer Diagnosis. NPJ Digit. Med..

[3] Wells A., Patel S., Lee J.B., Motaparthi K. (2021). Artificial Intelligence in Dermatopathology: Diagnosis, Education, and Research. J. Cutan. Pathol..

[4] Fliorent R., Engelman D., Guo C.Y., Tran T.A., Okoye G.A., Oaku I.K. (2024). Artificial Intelligence in Dermatology: Advancements and Challenges in Skin of Color. Int. J. Dermatol..

[5] Behara K., Bhero E., Agee J.T. (2024). AI in Dermatology: A Comprehensive Review into Skin Cancer Detection. PeerJ Comput. Sci..

[6] Daneshjou R., Vodrahalli K., Novoa R.A., Jenkins M., Liang W., Rotemberg V., Ko J., Swetter S.M., Bailey E.E., Gevaert O. (2022). Disparities in Dermatology AI Performance on a Diverse, Curated Clinical Image Set. Sci. Adv..

[7] Giavina-Bianchi M., Santos A.P., Cordioli E. (2020). Teledermatology Reduces Dermatology Referrals and Improves Access to Specialists. EClinicalMedicine.

[8] Nikolakis G., Brunner M., Engelbrecht D., Gasteratos K., Sievert H., Zouboulis C.C. (2024). Insights, Advantages, and Barriers of Teledermatology vs. Face-to-Face Dermatology for the Diagnosis and Follow-Up of Non-Melanoma Skin Cancer: A Systematic Review. Cancers.

[9] Wang R.H., Barbieri J.S., Nguyen H.P., Stavert R., Forman H.P., Bolognia J.L., Kovarik C.L., Group Y.D. (2020). Clinical Effectiveness and Cost-Effectiveness of Teledermatology: Where Are We Now, and What Are the Barriers to Adoption?. J. Am. Acad. Dermatol..

[10] Giavina-Bianchi M., Santos A.P., Cordioli E. (2020). Benefits of Teledermatology for Geriatric Patients: Population-Based Cross-Sectional Study. J. Med. Internet Res..

[11] Jiang S.W., Flynn M.S., Kwock J.T., Nicholas M.W. (2022). Store-and-Forward Images in Teledermatology: Narrative Literature Review. JMIR Dermatol..

[12] Jiang S.W., Flynn M.S., Kwock J.T., Nicholas M.W. (2022). Quality and Perceived Usefulness of Patient-Submitted Store-and-Forward Teledermatology Images. JAMA Dermatol..

[13] Zhou J., He X., Sun L., Xu J., Chen X., Chu Y., Zhou L., Liang L., Zhang B. (2024). Pre-Trained Multimodal Large Language Model Enhances Dermatological Diagnosis Using SkinGPT-4. Nat. Commun..

[14] Gui H., Rezaee M., Bhatt V., Gelfand J.M. (2024). The Promises and Perils of Foundation Models in Dermatology. J. Investig. Dermatol..

[15] Yan S., Yu Z., Zhang Q., Liu S., Zhang J., Xu X., Li X. (2025). A Multimodal Vision Foundation Model for Clinical Dermatology. Nat. Med..

[16] Hartsock I., Rasool G. (2024). Vision-Language Models for Medical Report Generation and Visual Question Answering: A Review. Front. Artif. Intell..

[17] Ryu J.S., Park J.E., Lee H., Kim D.W., Lee J.H. (2025). Vision-Language Foundation Models for Medical Imaging: A Review of Current Practices and Innovations. Biomed. Eng. Lett..

[18] Kvorning Ternov N., Nissen C.V., Taudorf E.H., Philipsen P.A., Wulf H.C. (2022). Generalizability and Usefulness of Artificial Intelligence for Skin Cancer Diagnostics: An Algorithm Validation Study. JEADV Clin. Pract..

[19] Rao P., Reese S., Gu K., Yun J., Liang L., Bui P., Garrett J., Kanzaria H., Liu Y. (2024). SCIN: A New Resource for Representative Dermatology Images.

[20] R Core Team (2025). R: A Language and Environment for Statistical Computing.

[21] Wickham H. (2016). ggplot2: Elegant Graphics for Data Analysis.

[22] Karampinis E., Zoumpourli C.M., Kontogianni C., Arkoumanis T., Koumaki D., Mantzaris D., Filippakis K., Papadopoulou M.M., Theofili M., Enechukwu N.A. (2026). Dermatology “AI Babylon”: Cross-Language Evaluation of AI-Crafted Dermatology Descriptions. Medicina.

