# Supplementary material for: Validation of a Dermatology-Focused Multimodal Image-and-Data Assistant in Diagnosis and Management of Common Dermatologic Conditions
Source: Medicina (Kaunas). 2026 Apr 9;62(4):715. doi: 10.3390/medicina62040715 (PMC13117915; doi:10.3390/medicina62040715)
Supplement: Supplementary file 1 [file medicina-62-00715-s001.zip › medicina-4231374-supplementary.pdf]

SUPPLEMENTARY MATERIALS

Validation of a Dermatology-Focused Multimodal Image-and-Data Assistant in Diagnosis and Management of Common Dermatologic Conditions

SUPPLEMENTARY PROTOCOL

1. Study Design Overview

This retrospective comparative study evaluated two artificial intelligence systems for diagnosis and management planning of inflammatory dermatoses (rashes) and neoplastic conditions: (1) DermFlow, a proprietary dermatology-focused system that autonomously collects patient history and analyzes images, and (2) Claude Sonnet 4 (Claude), Anthropic's state-of-the-art vision-language model that analyzes images without autonomously collecting additional patient history. Claude was selected as the comparator to establish an image-only baseline performance. Both systems were evaluated on identical clinical cases to compare diagnostic accuracy and management plan quality across diverse skin tones.

2. Study Population and Case Selection

2.1. Total Study Population

The study included 87 clinical images representing 10 common inflammatory dermatologic conditions (rashes) and 9 neoplastic dermatologic conditions stratified across three Fitzpatrick Skin Tone (FST) categories:

- FST I-II (lighter skin tones): 29 images (2 per rash diagnosis, 1 per neoplasm)
- FST III-IV (medium skin tones): 29 images (2 per rash diagnosis, 1 per neoplasm)
- FST V-VI (darker skin tones): 29 images (2 per rash diagnosis, 1 per neoplasm).

Images of rashes were obtained from the Skin Condition Image Network (SCIN) database [1], and images of neoplasms were obtained from the Diverse Dermatology Images (DDI) database [2]. Both databases are publicly available repositories of dermatologic images with diverse skin tone representation. The sample size was determined based on several considerations:

1. Representation of FST diversity: Equal distribution across FST categories (29 images each) was prioritized to enable robust analysis of equity in AI performance across skin tones.
2. Clinical diversity: Inclusion of 10 distinct inflammatory dermatoses and 9 neoplastic conditions provided a breadth of dermatologic presentations commonly encountered in clinical practice.

3. Resource constraints: Availability of sufficient numbers of images in the database for inclusion of a diagnosis in the study limited sample size.

2.2. Diagnosis Selection

10 common inflammatory dermatologic conditions were selected to represent a diverse range of rash and neoplastic presentations frequently encountered in clinical practice. These conditions were chosen because they (1) were sufficiently represented across FST categories within the SCIN and DDI databases and (2) may require integration of clinical history with visual assessment for accurate diagnosis, making them ideal test cases for evaluating multimodal AI approaches. The 10 included rash diagnoses were: allergic contact dermatitis, atopic dermatitis, folliculitis, herpes simplex, impetigo, insect bite, irritant contact dermatitis, psoriasis, tinea versicolor, and urticaria. The 9 included neoplastic diagnoses were: acrochordon, dermatofibroma, Kaposi sarcoma, melanocytic nevus, mycosis fungoides, nevus lipomatosus, seborrheic keratosis, squamous cell carcinoma, and verruca vulgaris.

3. Standardized Patient Histories

3.1. History Development

Standardized patient histories representative of each diagnosis were developed and presented in a question-and-answer format. These clinical vignettes included chief complaint, lesion duration, evolution over time, attempted treatments, clinical symptoms (including pertinent positive symptoms and pertinent negatives), alleviating and exacerbating factors, chronic conditions, current medications, allergies, family history, travel history, close contacts, recent exposures, sunscreen use, sun exposure, and psychosocial impact. Identical histories were used across all FST groups for the same diagnosis to ensure consistent clinical context.

3.2. History Presentation Format

Patient histories were presented to DermFlow in a question-and-answer format that simulated an interactive clinical encounter. DermFlow autonomously formulated questions to gather relevant history, and responses were provided only when specifically requested by the system. This approach allowed DermFlow to demonstrate its capability for targeted, adaptive history-taking while ensuring consistent information across all cases.

Patient histories were not presented to Claude. Claude received only the clinical images without any accompanying patient history, demographic information, or clinical context. This image-only approach represented the experimental control condition, allowing direct assessment of whether the addition of autonomously collected structured patient history (DermFlow) provided diagnostic and management advantages over image-based evaluation alone (Claude).

4. AI Model Evaluation Methodology

Each model was instructed to output a maximum of 4 differential diagnoses, ranked by likelihood, that were determined to have >85% likelihood. In addition, each model was allowed to provide an additional 1-2 diagnoses that are potentially life-threatening, highly morbid, rapidly progressive, or with potential systemic or other organ involvement (safety diagnoses), even if they were determined to have <85% likelihood. The correct diagnosis was then selected, and a comprehensive management plan was generated based on the correct diagnosis. The management plan included (1) diagnostic, (2) therapeutic, (3) counseling, and (4) monitoring recommendations. If the correct diagnosis was not included within the differential diagnosis of either DermFlow or Claude, the correct diagnosis was subsequently provided to the model prior to generation of the management plan.

5. Outcome Measures

5.1. Primary Outcome: Diagnostic Accuracy

Diagnostic accuracy was defined as the proportion of cases where the correct diagnosis appeared anywhere within the model's differential diagnosis list (including both primary likelihood-based diagnoses and safety diagnoses). For each case, diagnostic accuracy was coded as 1 (correct diagnosis present in differential) or 0 (correct diagnosis absent from differential). Overall accuracy was calculated as:

$$Diagnostic\ Accuracy = \frac{\#\ cases\ with\ correct\ diagnosis\ in\ differential}{total\ \#\ of\ cases}$$

5.2. Secondary Outcome: Management Plan Quality

Management plans generated by both AI systems were evaluated by two independent, board-certified dermatologists who were blinded to the model source. Each management plan was assessed using eight Likert-scale questions rated from 1 (poor/inappropriate) to 5 (excellent/highly appropriate). Evaluation criteria can be found in Table 1 of the main manuscript.

Each domain was rated on a 5-point Likert scale, where 1 = Poor/Inappropriate (major concerns, unsafe, or ineffective), 2 = Fair (significant limitations or safety concerns), 3 = Good (acceptable with minor improvements needed), 4 = Very Good (high quality with minimal limitations), and 5 = Excellent (optimal management, highly appropriate)

For each management plan, mean scores were calculated across all eight domains. The maximum possible score was 5.0 (all domains rated 5), and the minimum possible score was 1.0 (all domains rated 1). Individual domain scores and overall mean scores were analyzed separately. While no predefined threshold was established to categorize management plans as "poor quality," scores <3.0 indicated management plans with significant limitations requiring improvement, and scores ≥4.0 indicated high-quality management suitable for clinical implementation.

6. Statistical Analysis

6.1. Statistical Tests

Diagnostic accuracy was compared between models using Pearson chi-square tests, without continuity correction, for overall differences and within FST strata. When expected cell counts were <5, Fisher's exact tests were used to verify validity of chi-square results. Accuracy rates are reported as percentages with 95% confidence intervals calculated using the normal approximation method. Management plan quality scores were compared using independent samples t-tests for overall comparisons and analysis of variance (ANOVA) with Tukey post-hoc testing for FST-stratified analyses. Two-sided p-values < 0.05 were considered statistically significant. To control for multiple comparisons across 59 hypothesis tests, we applied the Benjamini-Hochberg procedure with a false discovery rate of 5%. P-values were ranked in ascending order, and critical values were calculated as $(i/59) \times 0.05$, where i is the p-value's rank. The largest p-value below its critical value and all smaller p-values were considered. All analyses were performed using R version 4.5.1 and figures were generated with ggplot2 package [3,4].

6.2. Power Analysis for Overall Diagnostic Accuracy

A post-hoc power analysis was conducted for the primary outcome (diagnostic accuracy comparison between DermFlow and Claude) using an exact McNemar framework.

Parameters:

- Sample size: 87 paired observations
- Number of discordant pairs: 56 (55 DermFlow-correct/Claude-incorrect and 1 DermFlow-incorrect/Claude-correct)
- Significance level: $\alpha = 0.05$ (two-tailed)

Sensitivity analyses showed adequate power to detect moderate-to-large discordant imbalances (e.g., ~86% power for a 70/30 split and ~97% for a 75/25 split) at $\alpha = 0.05$ (two-tailed).

6.3 Power Analysis for FST Comparisons of Diagnostic Accuracy

A post-hoc sensitivity-based power analysis was conducted for within-model comparisons of diagnostic accuracy across 3 FST categories using an exact McNemar framework

Parameters:

- Sample size: 29 paired observations per FST category
- Number of discordant pairs: 18-20 per FST category
- Significance level: $\alpha = 0.05$ (two-tailed)

Sensitivity analyses demonstrated limited power for moderate discordant imbalances (e.g., ~52-62% power for a 75/25 split). However, the observed discordant splits (18/0, 20/0, and 17/1)

were substantially larger than moderate thresholds and achieved power under the exact McNemar framework was effectively complete in each stratum.

6.4 Power Analysis for Overall Management Quality

A post hoc simulation-based power analysis was performed for management quality outcomes using Wilcoxon rank-sum and Kruskal-Wallis frameworks, reflecting the nonparametric tests used in the primary analyses.

Our management plan quality comparisons considered:

- 87 cases per model
- 2 reviewers per case (174 observations per model)
- 7-8 data points per observation (approximately 1300 data points per model)
- Significance level: $\alpha = 0.05$

Based on the observed number of rating observations and score variability, the study had high power to detect small and moderate between-model differences in management quality.

- For differences of 0.2-0.5 points: >99% power
- For differences of 0.1 points: 65.9% power.

Based on the observed number of rating observations and score variability, the study had high power to detect small-to-moderate and moderate within-model differences in management quality related to FST category.

- For differences of 0.3-0.5 points: >98% power
- For differences of 0.2 points: 77.2–78.7% power
- For differences of 0.1 points: 27.6–28.4% power.

References

1. Rao P, Reese S, Gu K, et al. SCIN: A new resource for representative dermatology images. Google Research; 2024.
2. Daneshjou R, Vodrahalli K, Novoa RA, Jenkins M, Liang W, Rotemberg V, Ko J, Swetter SM, Bailey EE, Gevaert O, Mukherjee P, Phung M, Yekrang K, Fong B, Sahasrabudhe R, Allerup JAC, Okata-Karigane U, Zou J, Chiou AS. Disparities in dermatology AI performance on a diverse, curated clinical image set. Sci Adv. 2022 Aug 12;8(32):eabq6147. doi: 10.1126/sciadv.abq6147. Epub 2022 Aug 12. PMID: 35960806; PMCID: PMC9374341.
3. Team, R.C., R: A language and environment for statistical computing. 2025, R Foundation for Statistical Computing: Vienna, Austria.
4. Wickham, H., ggplot2: Elegant Graphics for Data Analysis. 2016, Springer-Verlag: New York, NY.

Table S1. Questions and examples considered by dermatologist reviewers when rating management plans.

Rating Category	Question	Examples
Appropriateness	How clinically appropriate is this management plan?	Incorrect facts about the condition; inadequate time frames for medication regimens; inappropriate choice, usage, or dosage of first-line therapy.
Safety	How safe is this management plan for the patient?	Recommending systemic immunosuppression without baseline laboratory monitoring; failing to mention drug interactions or contraindications; omitting pregnancy considerations for teratogenic medications such as methotrexate.
Usefulness	How useful would this management plan be for a dermatologist in clinical practice?	Providing vague recommendations without specific drug names, dosages, or durations; including overly generic advice that does not differentiate from information readily available to the patient online.
Risk Screening	How well does the management plan account for appropriate screening of associated conditions (if applicable)?	Screening for psoriatic arthritis in a psoriasis diagnosis; referral for a full skin exam in a melanoma diagnosis; metabolic syndrome screening with multiple acrochordons.
Diagnostic Workup	Overall rating of AI diagnostic workup plan.	Recommending biopsy when clinical diagnosis is sufficient; omitting dermoscopy

		for pigmented lesions; failing to suggest patch testing for suspected allergic contact dermatitis.
Treatment Plan	Overall rating of AI treatment plan.	Appropriate stepwise therapy starting with topical agents before systemic therapy; specifying vehicle, potency, and duration for topical corticosteroids; including both pharmacologic and non-pharmacologic options.
Counseling	Overall rating of AI counseling.	Explaining the chronic or relapsing nature of the condition; addressing common triggers and avoidance strategies; setting realistic expectations for treatment response and timeline.
Monitoring	Overall rating of AI monitoring plan.	Specifying appropriate follow-up intervals; including laboratory monitoring for systemic therapies; defining criteria for treatment escalation or specialist referral.

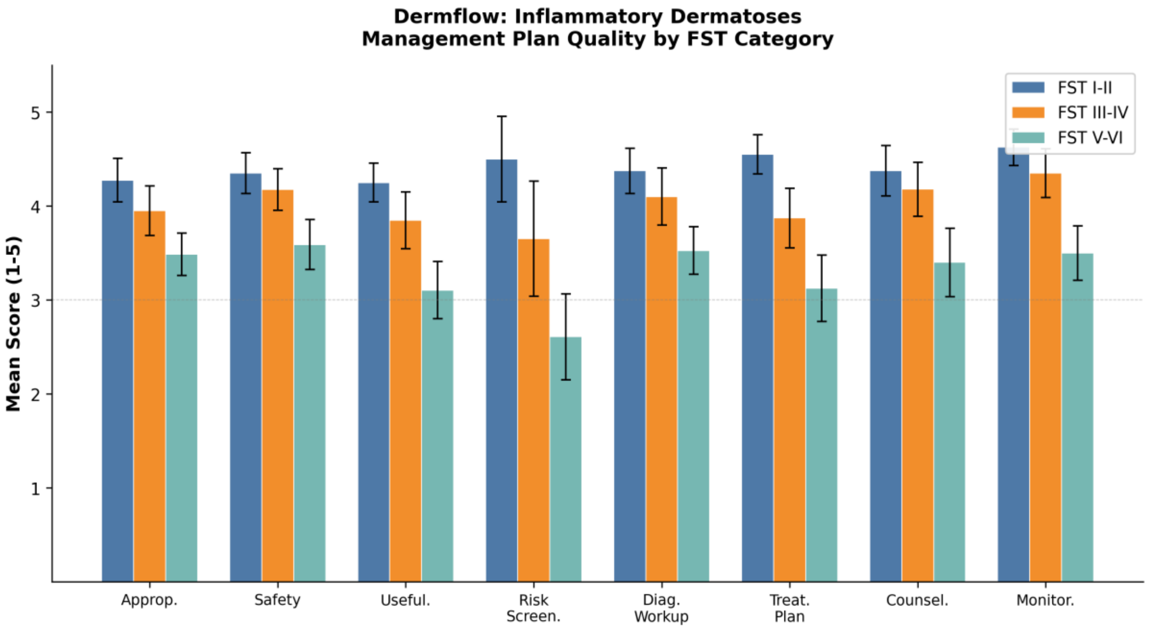

Figure S1. Management plan quality scores of Dermflow for inflammatory dermatoses, stratified by Fitzpatrick Skin Tone category. Error bars represent 95% confidence intervals.

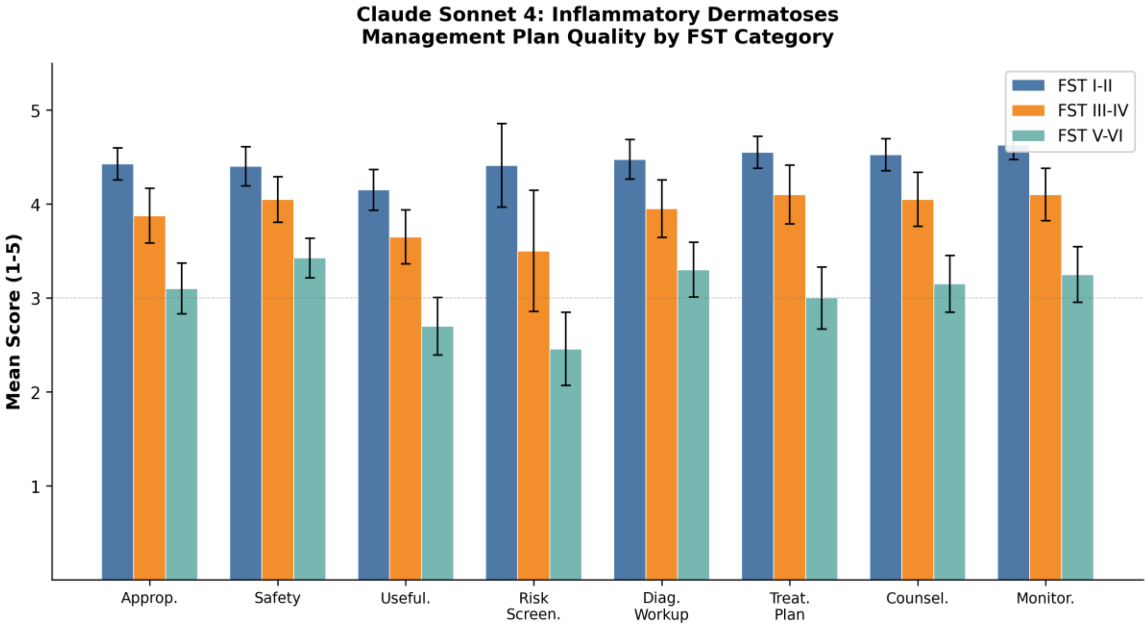

Figure S2. Management plan quality scores of Claude Sonnet 4 for inflammatory dermatoses, stratified by Fitzpatrick Skin Tone category. Error bars represent 95% confidence intervals.

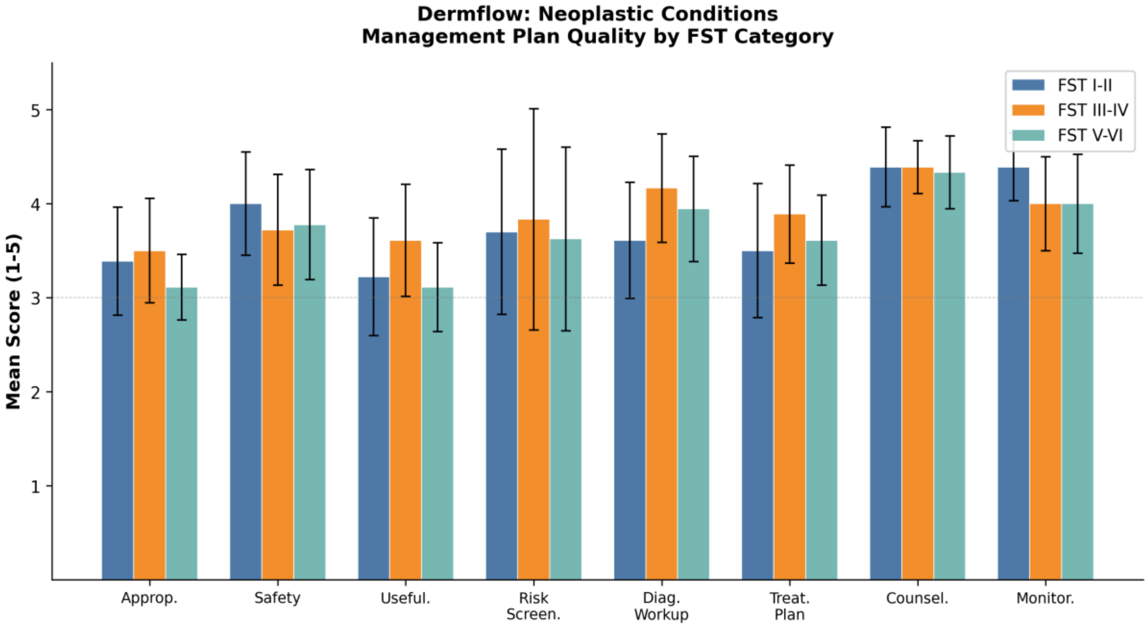

Figure S3. Management plan quality scores of Dermflow for neoplastic conditions, stratified by Fitzpatrick Skin Tone category. Error bars represent 95% confidence intervals.

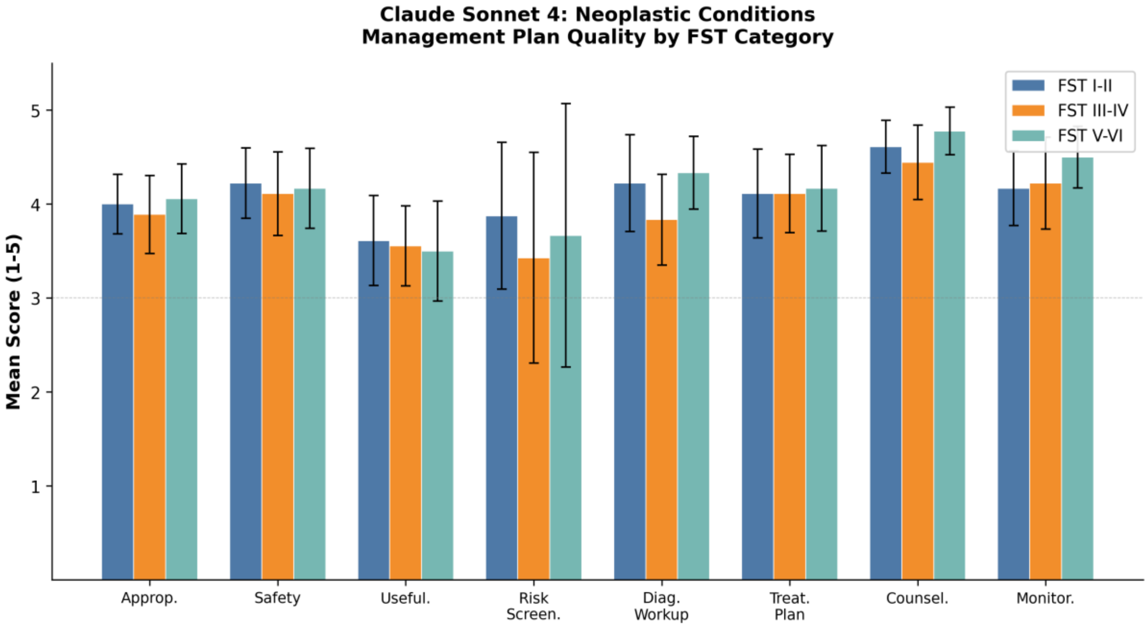

Figure S4. Management plan quality scores of Claude Sonnet 4 for neoplastic conditions, stratified by Fitzpatrick Skin Tone category. Error bars represent 95% confidence intervals.
